# Supplementary figures and images for: Alpha-herpesvirus US1 interacts with cGAS to suppress type I IFN responses and antiviral defense
Source: PLoS Pathog. 2025 Nov 6;21(11):e1013669. doi: 10.1371/journal.ppat.1013669 (PMC12604781; doi:10.1371/journal.ppat.1013669)

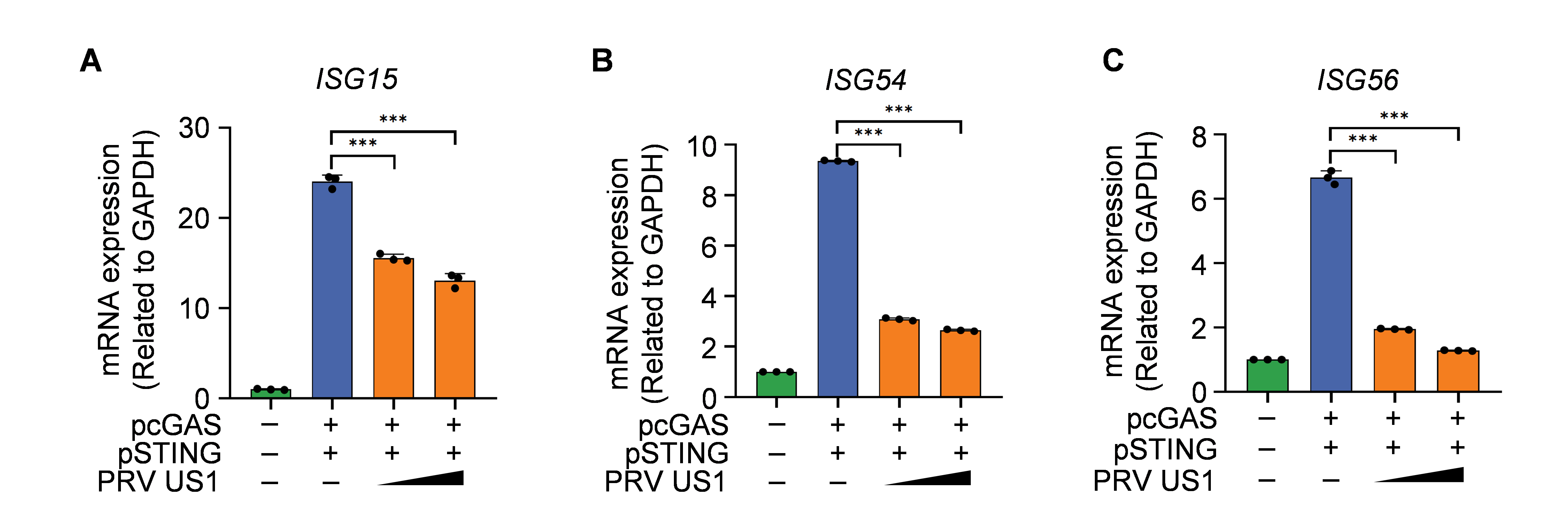

Supplement: S1 Fig — (A, B and C) HEK-293T cells were transfected with plasmids encoding pcGAS, pSTING, and increasing doses of PRV US1. At 24 hours post-transfection (hpt), cells were harvested for RNA extraction, followed by RT-PCR analysis of ISG15 (A), ISG54 (B), ISG56 (C) and GAPDH. Representative results from three biological replicates are shown. Data represent mean values ± SD of three technical replicates. Statistical significance was determined by two-tailed unpaired Student’s t-test. ***P < 0.0001. (TIF) [file ppat.1013669.s001.tif]

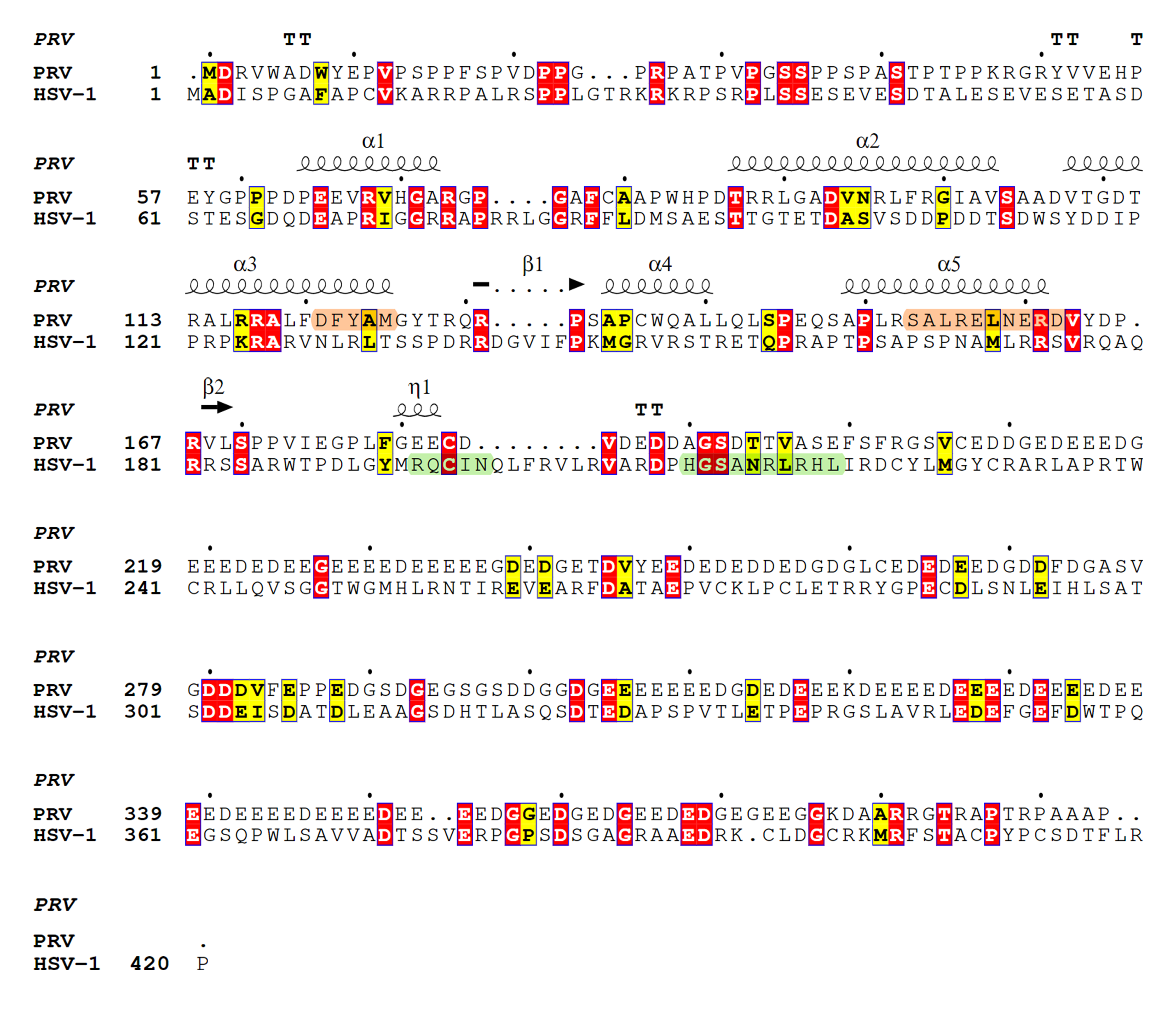

Supplement: S2 Fig — Identical residues were highlighted with a red background, while similar residues were highlighted with a yellow background. α-helices were represented as squiggles. β-strands were depicted as arrows, and strict β-turns were indicated by TT letters. (TIF) [file ppat.1013669.s002.tif]

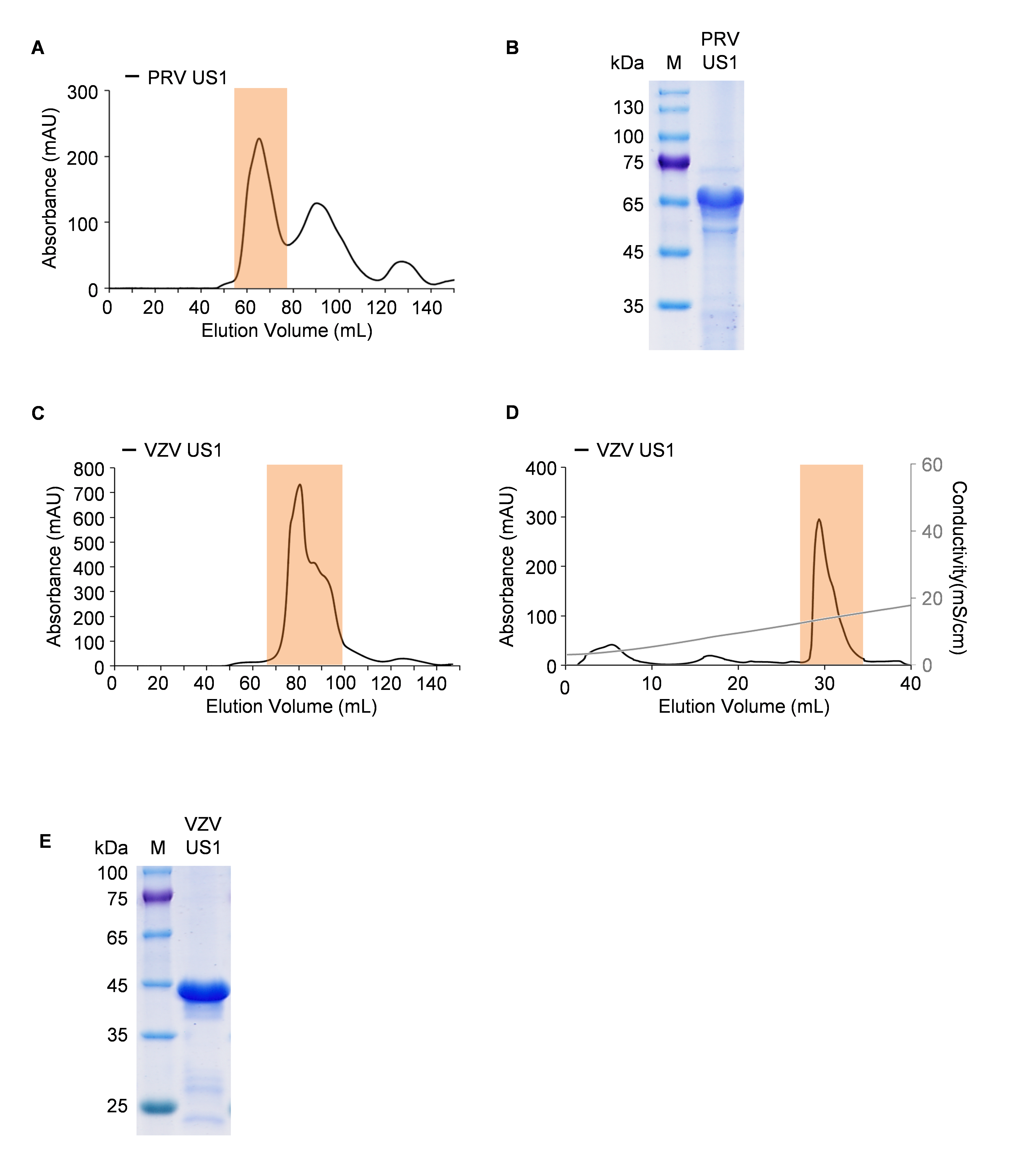

Supplement: S3 Fig — (A, B) PRV US1 recombinant proteins were purified using gel filtration (A). The SUMO tag of recombinant proteins was removed by SUMO protease treatment overnight at 4°C followed by SDS-PAGE analysis (B). (C-E) VZV US1 recombinant proteins were purified using gel filtration (C) and ion exchange (D). The SUMO tag of recombinant proteins was removed by SUMO protease treatment overnight at 4°C, followed by SDS-PAGE analysis (E). (TIF) [file ppat.1013669.s003.tif]

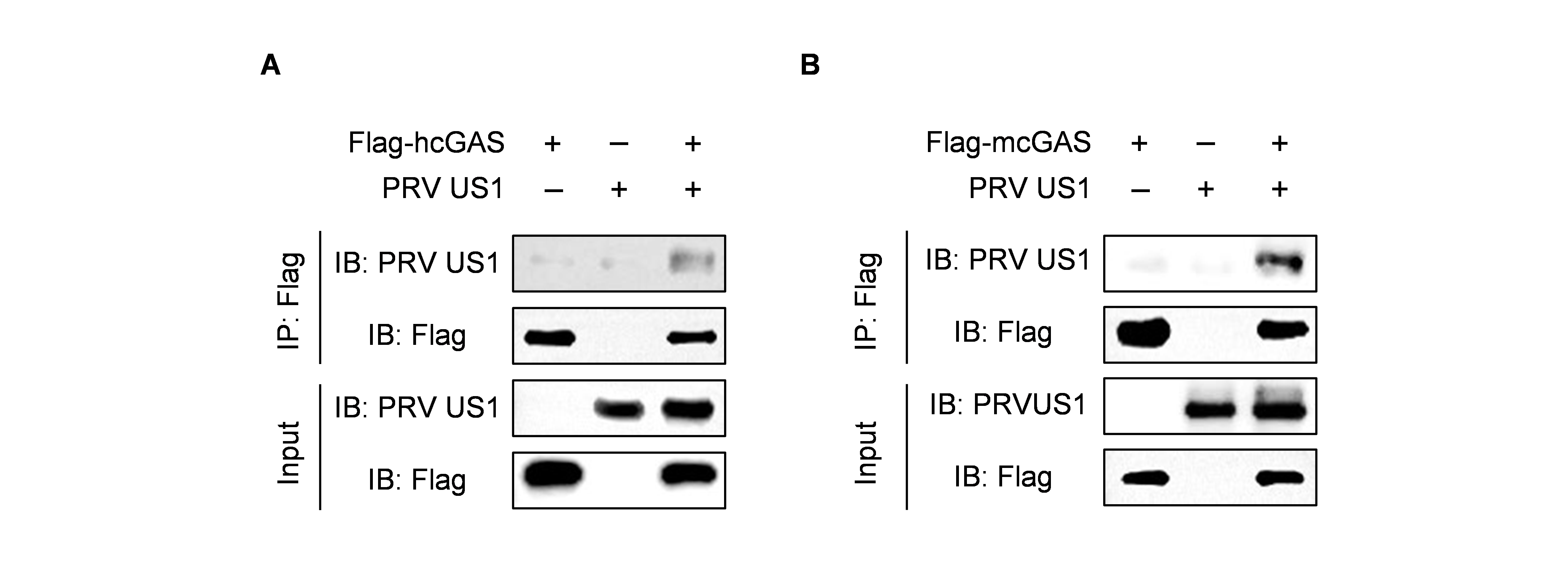

Supplement: S4 Fig — (A, B) Co-IP assay for assessment of the interactions between PRV US1 and hcGAS (A) or mcGAS (B). After mixing the purified recombinant proteins in IP buffer at 4 °C for 2 hours, the mixed buffer was immunoprecipitated using mouse anti-Flag MAb. The immunoprecipitated complexes were analyzed by immunoblotting with the indicated antibodies. Results are representative of three biological replicates. Representative results from three biological replicates are shown. (TIF) [file ppat.1013669.s004.tif]

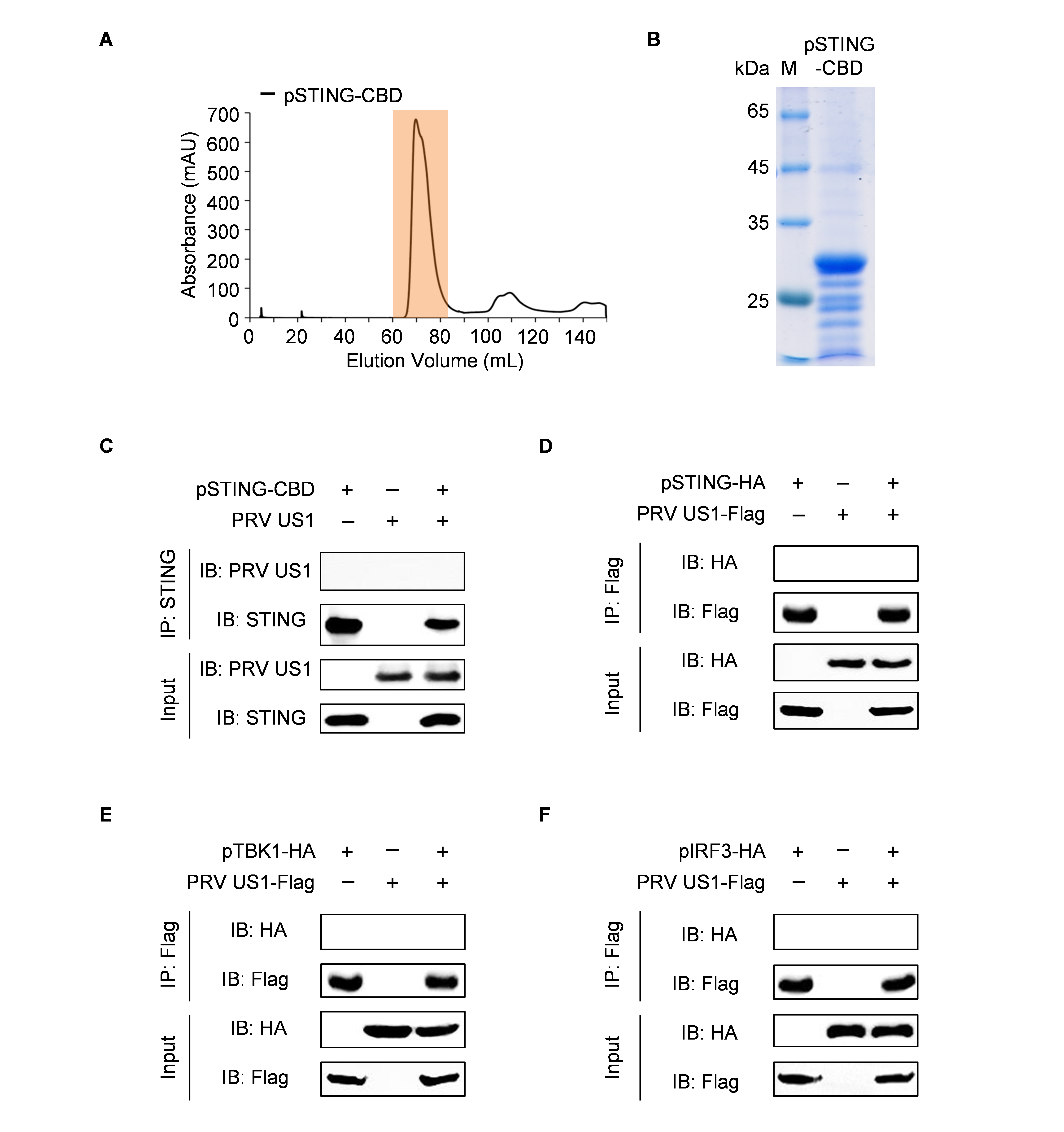

Supplement: S5 Fig — (A, B) Recombinant proteins pSTING-CBD were purified using gel filtration. The SUMO tag of recombinant proteins was removed by SUMO protease treatment overnight at 4°C. (C) Co-IP assay for the purified recombinant PRV US1 protein and pSTING-CBD protein. After mixing the purified recombinant proteins in IP buffer at 4 °C for 2 hours, the mixed buffer was immunoprecipitated using mouse anti-Flag MAb. The immunoprecipitated complexes were analyzed by immunoblotting with the indicated antibodies. (D-F) Co-IP assay for assessment of the interactions between PRV US1 and pSTING (D), pTBK1 (E) or pIRF3 (F) in HEK-293T cells. After 24 h post-transfection of plasmids expressing two target proteins, the cells were lysed, and the supernatants were treated like the mixed buffer in (C). (C-F) Representative results from three biological replicates are shown. (TIF) [file ppat.1013669.s005.tif]

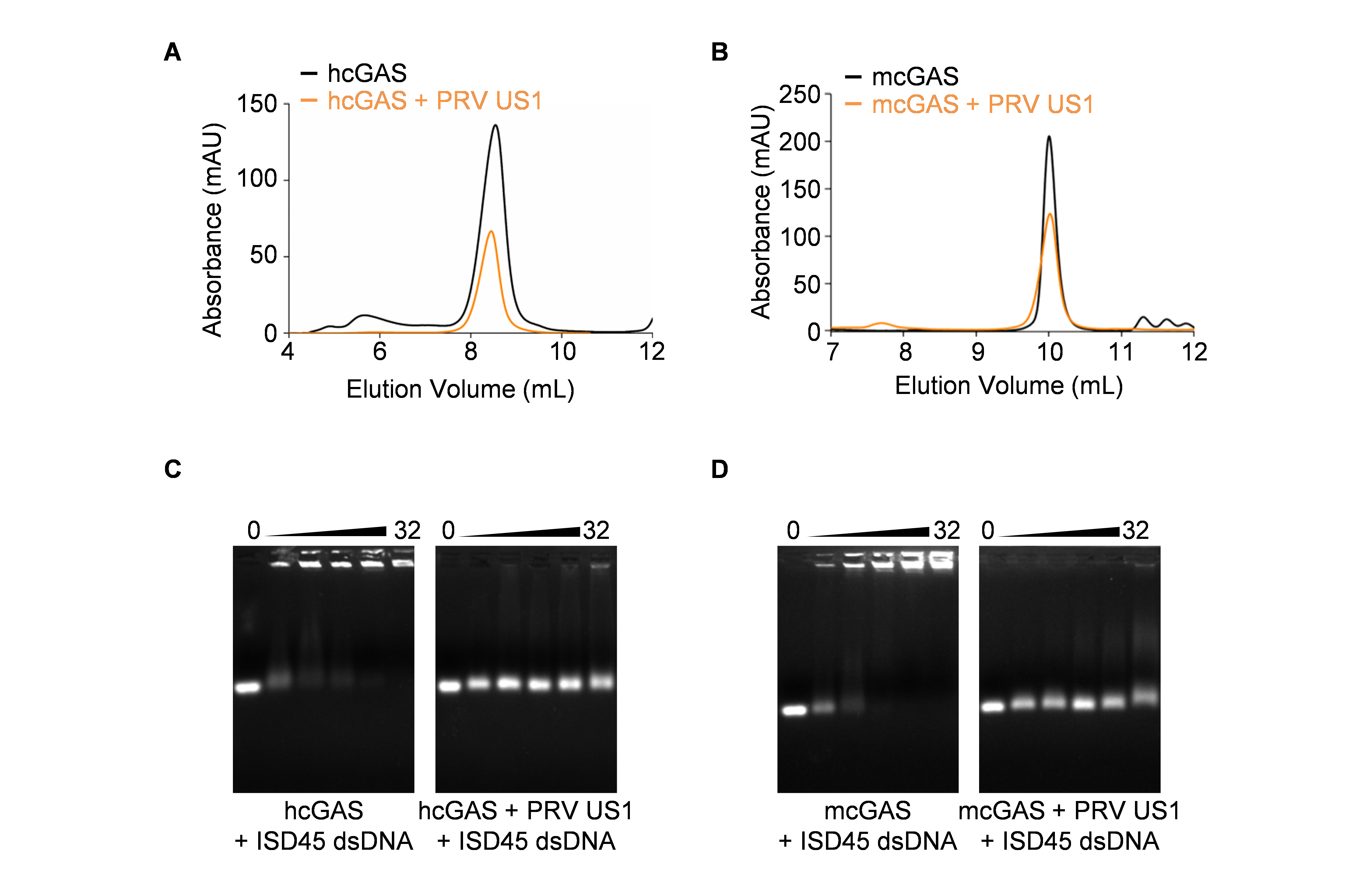

Supplement: S6 Fig — (A, B) cGAS activity assay using ion exchange chromatography. 10 μM hcGAS (A) or mcGAS (B) was incubated with the Salmon Sperm DNA and equimolar ratio PRV US1 proteins in reaction buffer at 37°C for 2 h. The reaction product was first purified by ultrafiltration and then analyzed using a MonoQ ion exchange column. (C, D) DNA binding analysis of hcGAS (E) or mcGAS (F) under the influence of PRV US1. In a mixture of 2.5 μM ISD45, US1 and cGAS, proteins were increased in a molar ratio of 1:0–1:32, followed by EMSA. (A-D) Representative results from three biological replicates are shown. (TIF) [file ppat.1013669.s006.tif]

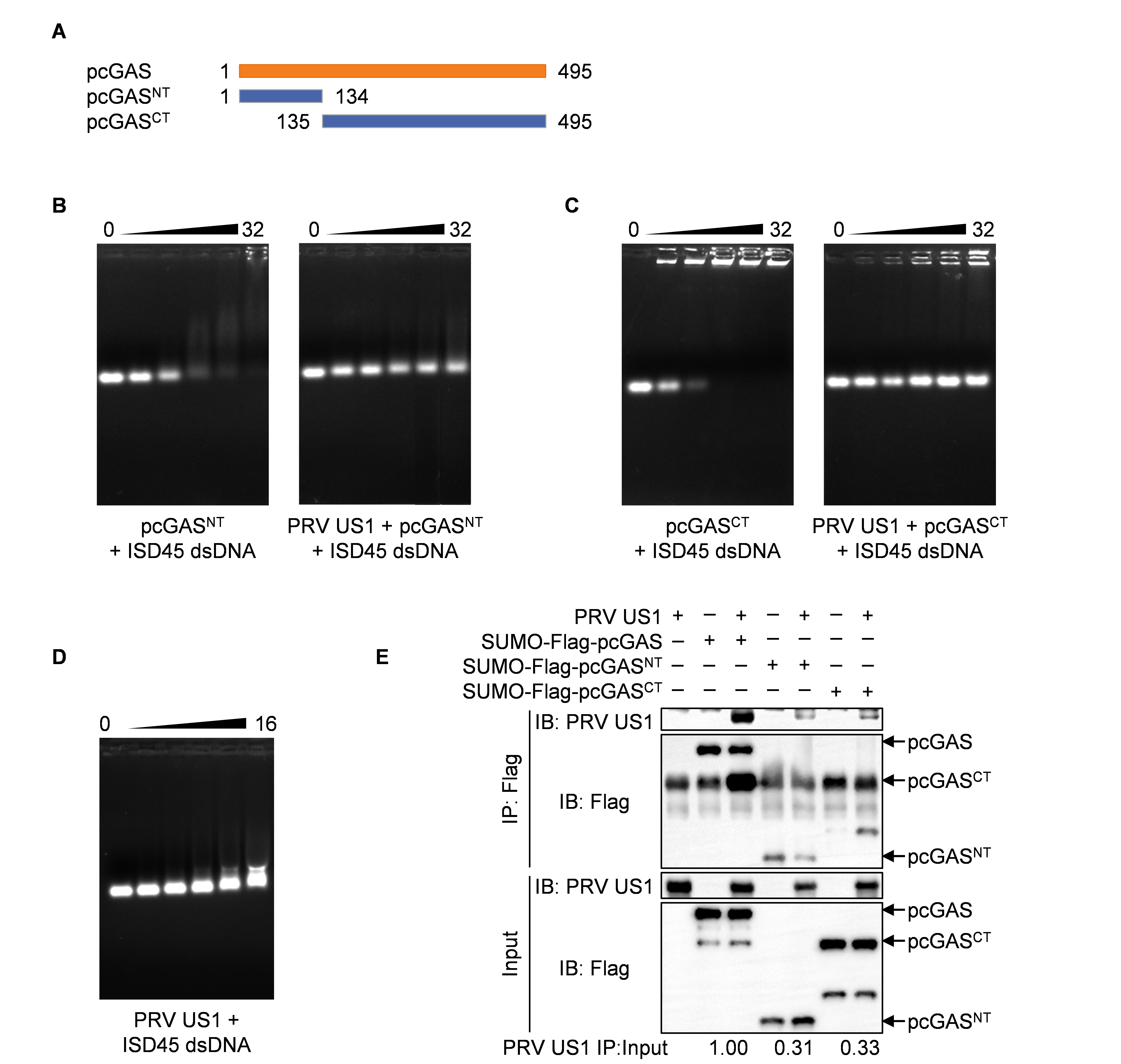

Supplement: S7 Fig — (A) Schematic diagram of pcGAS truncated domains. (B, C) DNA binding analysis of pcGASNT or pcGASCT under the influence of PRV US1. In a mixture of 2.5 μM 45 bp interferon stimulated DNA (ISD45), PRV US1 and pcGASNT (B) or pcGASCT (C), proteins were increased in a molar ratio of 1:0–1:32, followed by EMSA. (D) DNA binding analysis of PRV US1. The reaction system containing ISD45 and PRV US1 recombinant proteins with a molar ratio of 0–16 was treated as in (B). (E) Co-IP analysis to evaluate the interaction between PRV US1 and pcGAS or its truncation mutants. Purified recombinant proteins were mixed in IP buffer at 4°C for 2 hours, followed by immunoprecipitation of the mixture using mouse anti-Flag MAb. The immunoprecipitated complexes were analyzed by immunoblotting with the specified antibodies. Densitometric quantitation of PRV US1 were normalized relative to the levels of input by ImageJ. (B-E) Representative results from three biological replicates are shown. (TIF) [file ppat.1013669.s007.tif]

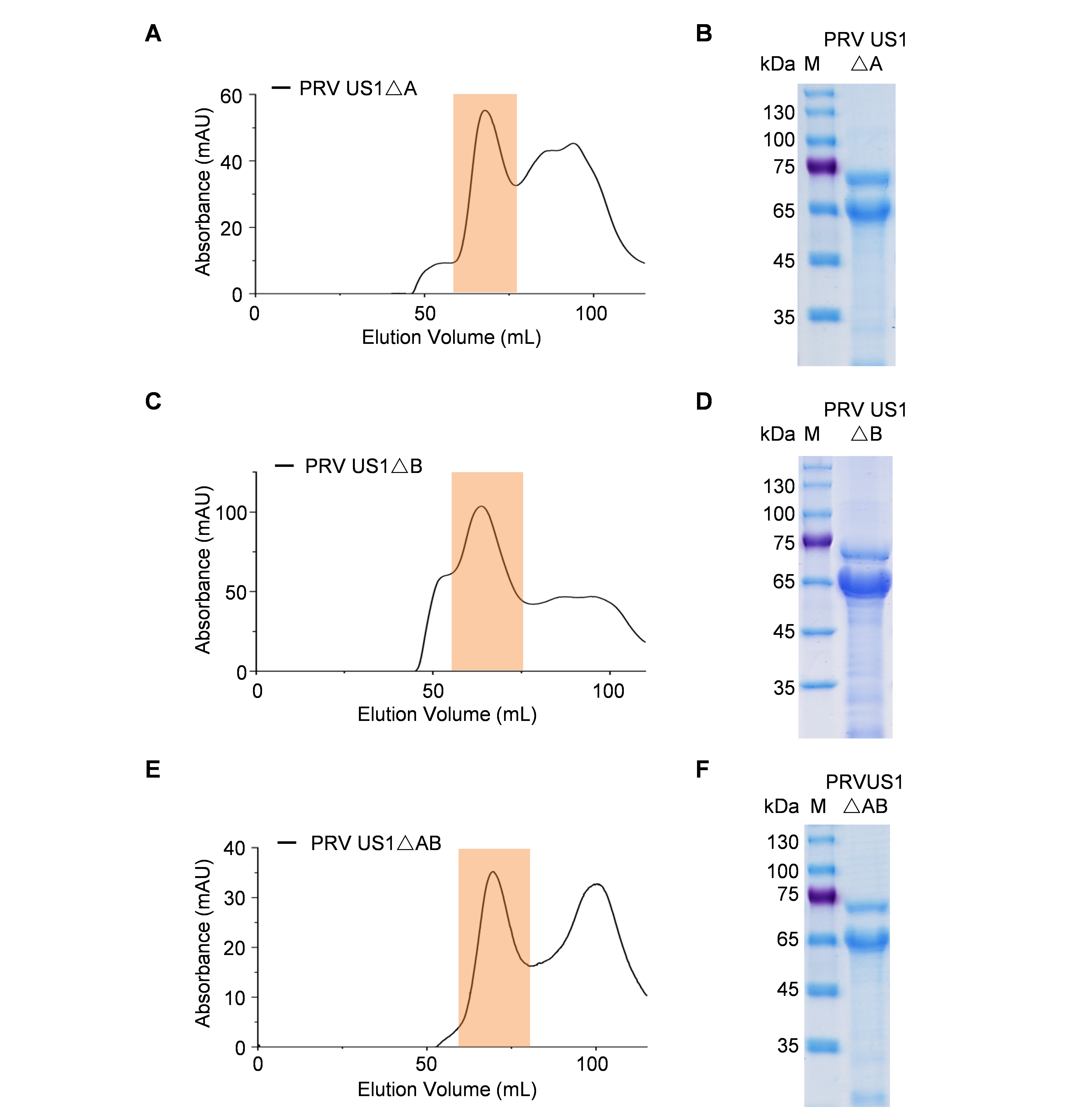

Supplement: S8 Fig — (A-F) The SUMO tag of recombinant SUMO-PRV US1 or its deletion mutant proteins was removed by SUMO protease treatment overnight at 4°C. The recombinant proteins PRV US1ΔA (A), PRV US1ΔB (C) and PRV US1ΔAB (E) were purified using gel filtration, followed by SDS-PAGE analysis (B, D and F). (TIF) [file ppat.1013669.s008.tif]

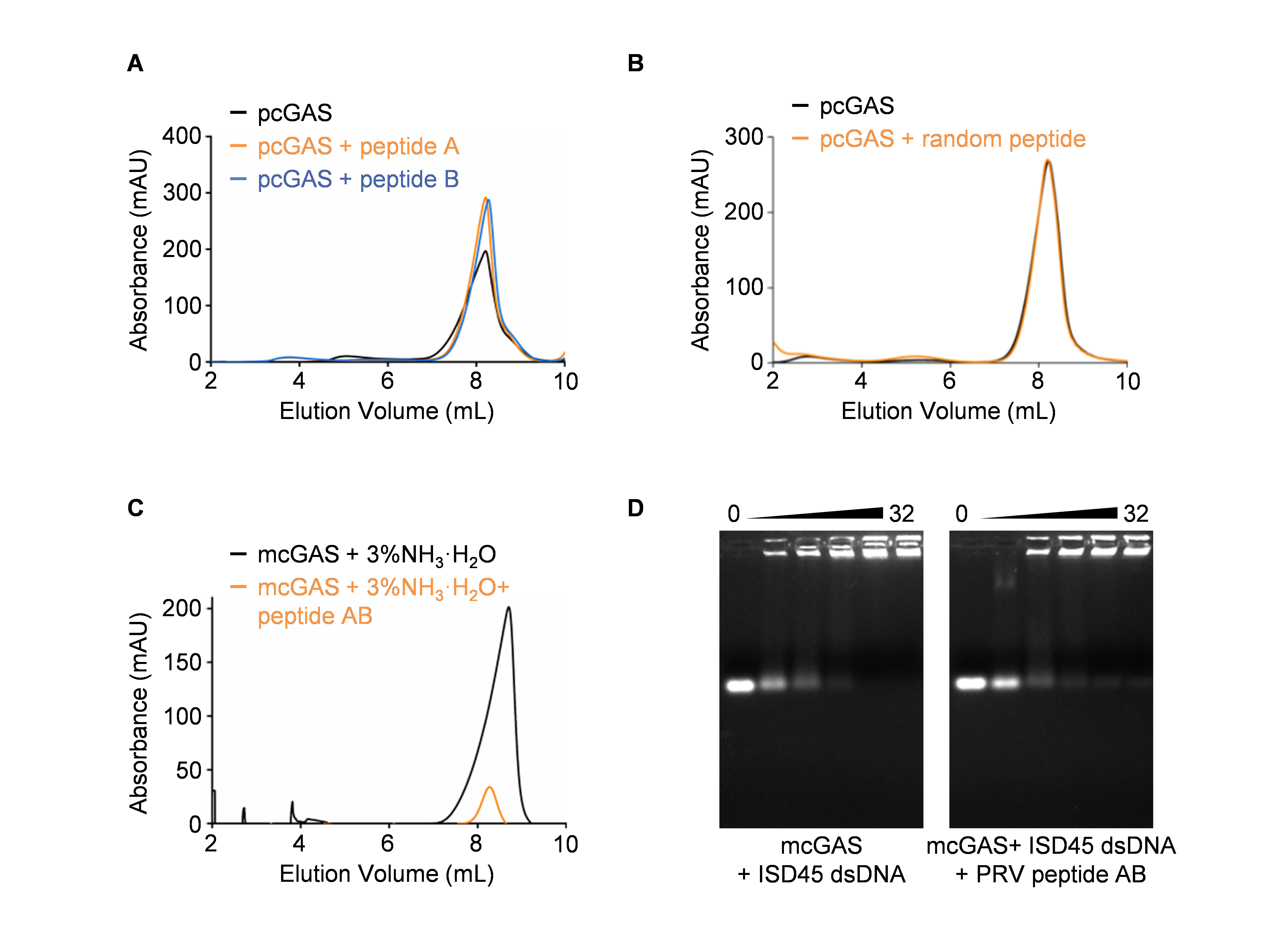

Supplement: S9 Fig — (A, B) cGAS activity assay by ion exchange chromatography. 10 μM pcGAS was incubated with the Salmon Sperm DNA and equimolar ratio peptide A, peptide B (A) or random peptide (B) in reaction buffer at 37°C for 2 h. The reaction product was first purified by ultrafiltration and then analyzed using a MonoQ ion exchange column. (C) Peptide AB was dissolved in 3% ammonia water, and then added to the enzymatic reaction system for mcGAS enzyme activity assay by ion exchange chromatography. cGAS activity assay was conducted as described in A, with an equal amount of ammonia water added as a control. (D) DNA binding analysis of mcGAS under the influence of PRV peptide AB. In a mixture of 2.5 μM ISD45, peptide and cGAS, peptide weas increased in a molar ratio of 1:0–1:32, followed by EMSA. (A-D) Representative results from three biological replicates are shown. (TIF) [file ppat.1013669.s009.tif]

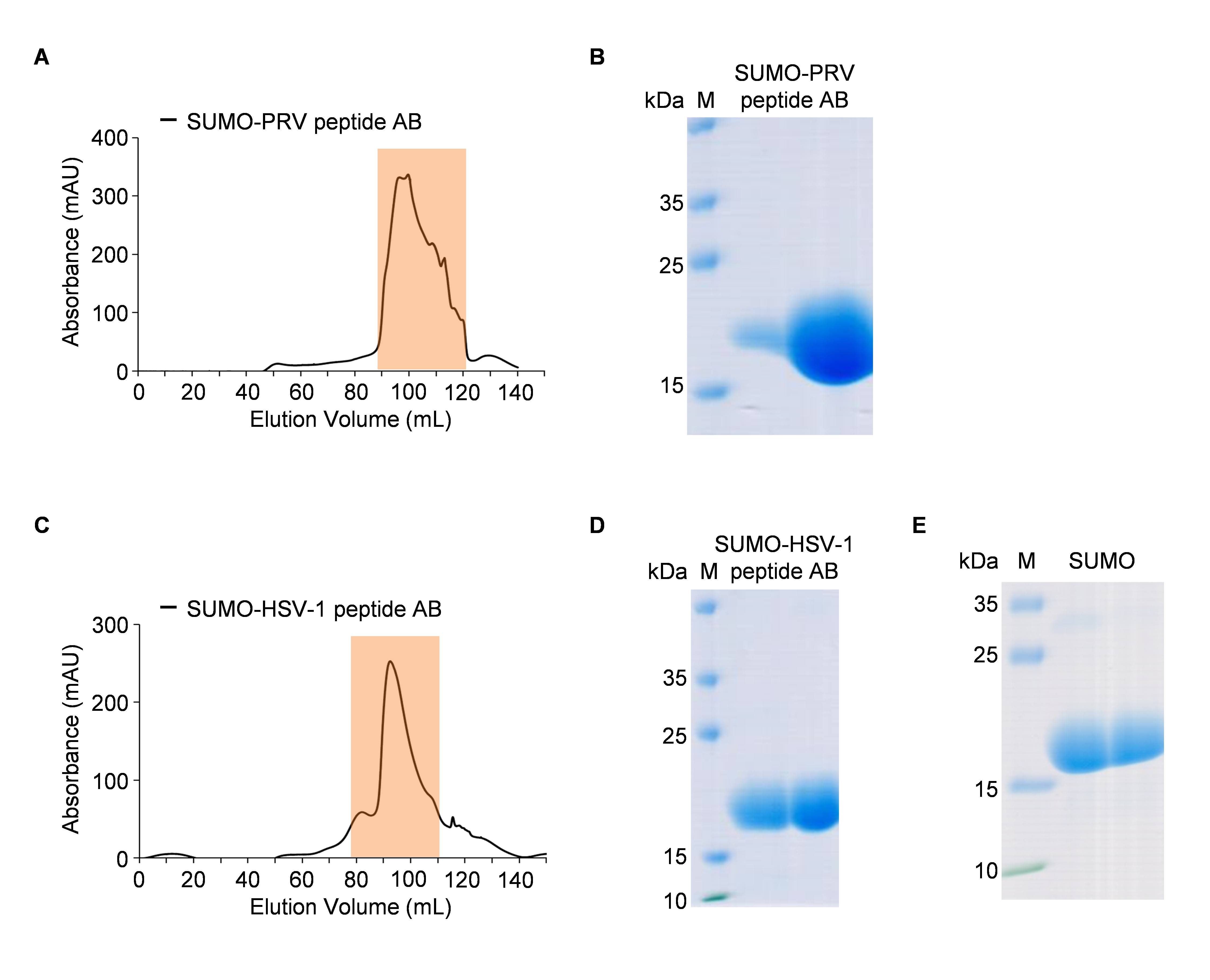

Supplement: S10 Fig — (A-D) The recombinant proteins SUMO-PRV US1 peptide AB (A) and SUMO-HSV-1 US1 peptide AB (C) were purified using gel filtration, followed by SDS-PAGE analysis (B and D). (E) Control SUMO proteins were obtained by overnight cleavage at 4°C using SUMO protease. (TIF) [file ppat.1013669.s010.tif]

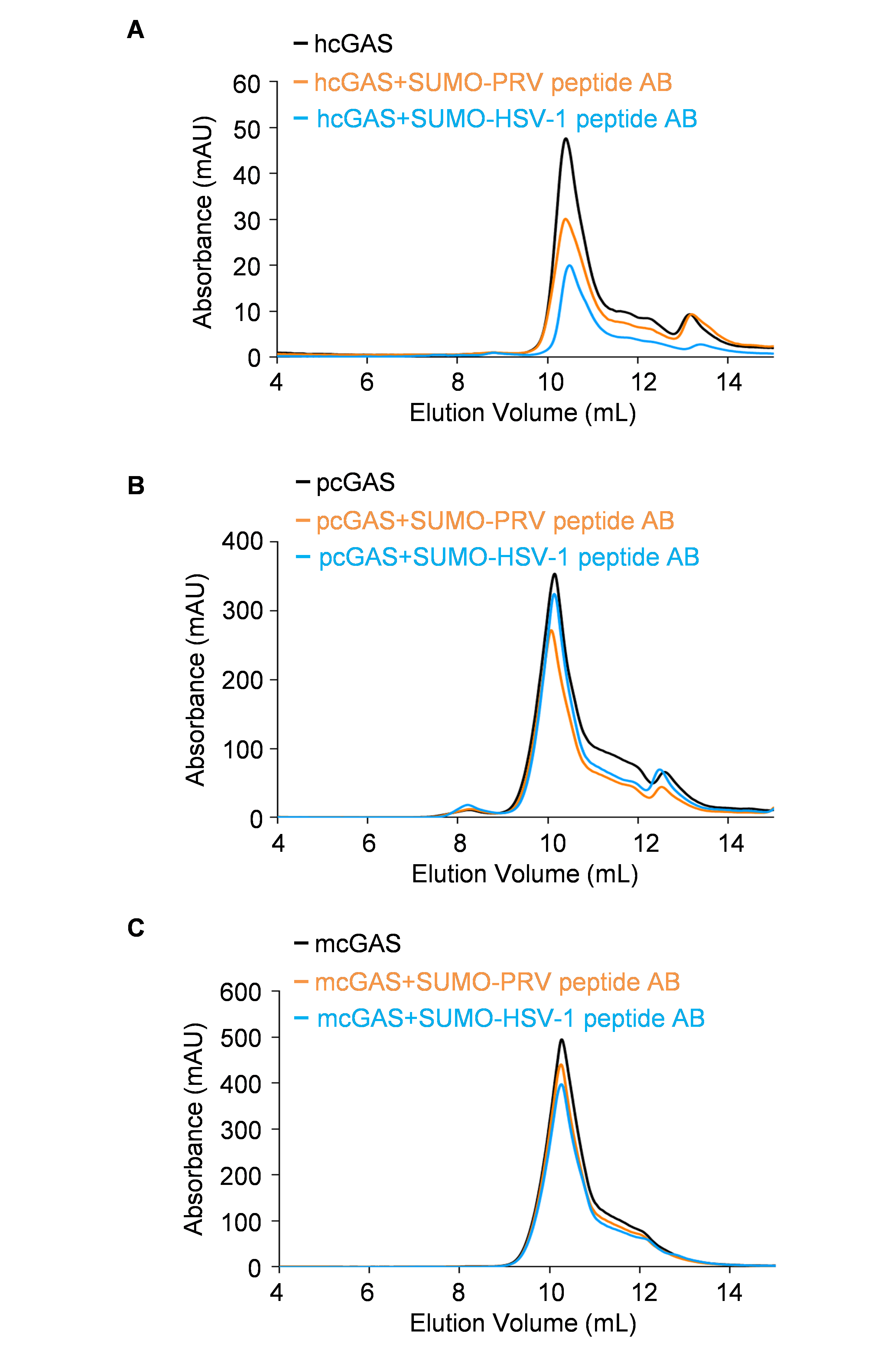

Supplement: S11 Fig — (A-C) cGAS activity assay using ion exchange chromatography. 10 μM hcGAS (A), pcGAS (B) or mcGAS (C) was incubated with the Salmon Sperm DNA and equimolar ratio SUMO-PRV peptide AB or SUMO-HSV-1 peptide AB in reaction buffer at 37°C for 2 h. The reaction product was first purified by ultrafiltration and then analyzed using a MonoQ ion exchange column. Results are representative of three biological replicates. Representative results from three biological replicates are shown. Representative results from three biological replicates are shown. (TIF) [file ppat.1013669.s011.tif]

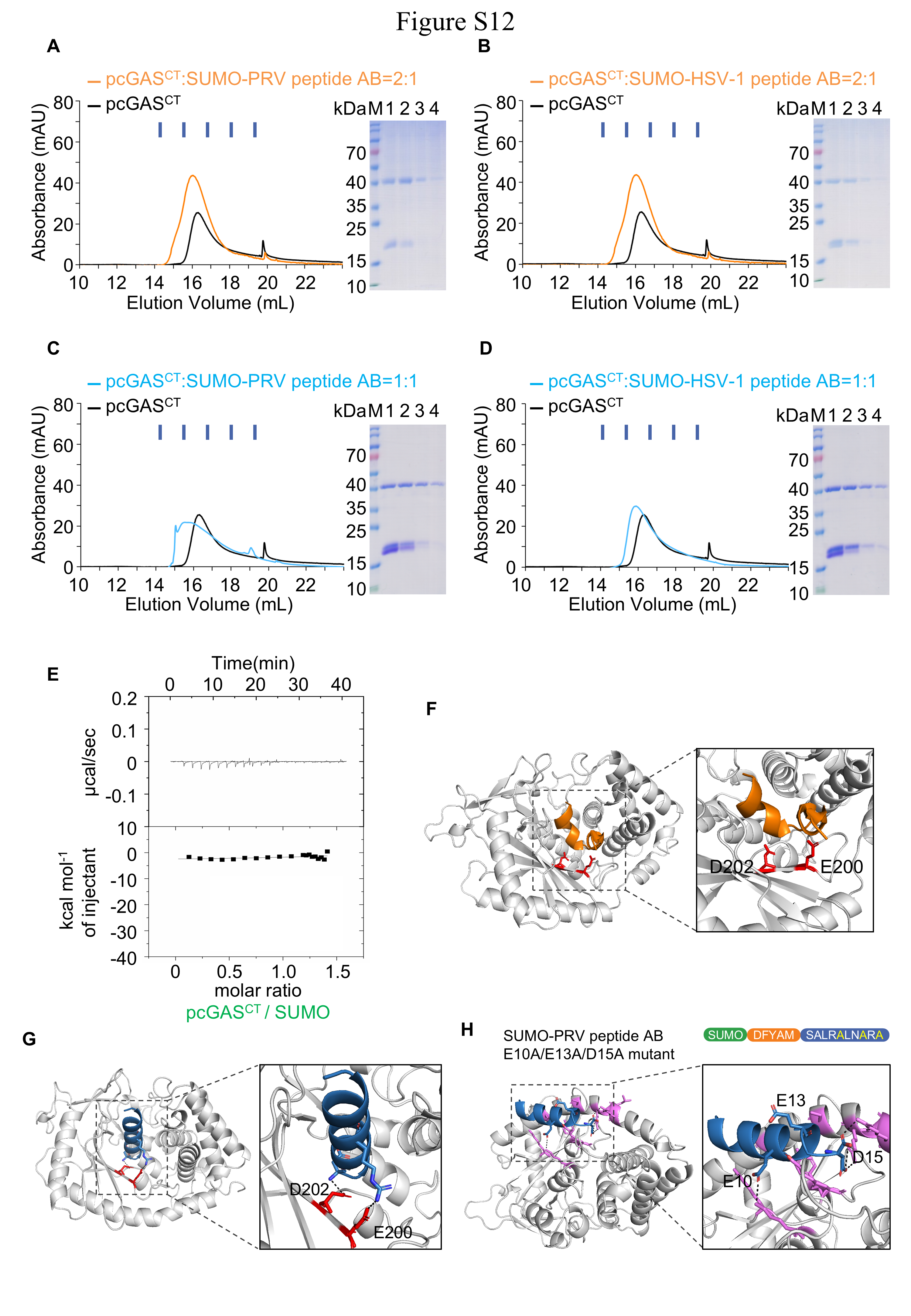

Supplement: S12 Fig — (A-D) Gel filtration chromatography analysis to determine whether pcGASCT binds to SUMO-PRV peptide AB (A, C) or HSV-1 peptide AB (B, D). pcGASCT was mixed with SUMO-PRV peptide AB or SUMO-HSV-1 peptide AB at a molar ratio of 2:1 or 1:1 and incubated on ice for 30 minutes. The samples were then analyzed by gel filtration chromatography. Blue lines above the gel filtration chromatogram denote fraction collection ranges for electrophoresis. The corresponding SDS-PAGE lanes (right) are labeled 1–4, corresponding to the first through fourth fractions indicated by blue lines. The shifted chromatographic peak with increased absorbance aligns with the presence of the proteins in the same fractions, confirming the formation of a protein complex. (E) The interaction between pcGAS and SUMO was analyzed using ITC. A 100 μM solution of pcGASCT was titrated into 300 μl of 10 μM SUMO solution, with 2 μl injected per titration. Data were collected at 25°C using a MicroCal ITC 200 isothermal titration calorimeter. (F) The complex structural model of HSV-1 peptide AB-pcGAS predicted by AlphaFold2. Yellow indicated HSV-1 peptide AB, gray indicated pcGASCT, and red indicated the enzyme active sites of pcGAS. (A-E) Representative results from three biological replicates are shown. (G, H) The complex structural models of PRV peptide AB-pcGAS predicted by AlphaFold2. Blue indicated PRV peptide AB, gray indicated pcGASCT, red indicated enzyme active sites of pcGAS, and pink indicated the DNA binding sites of pcGAS. (TIF) [file ppat.1013669.s012.tif]

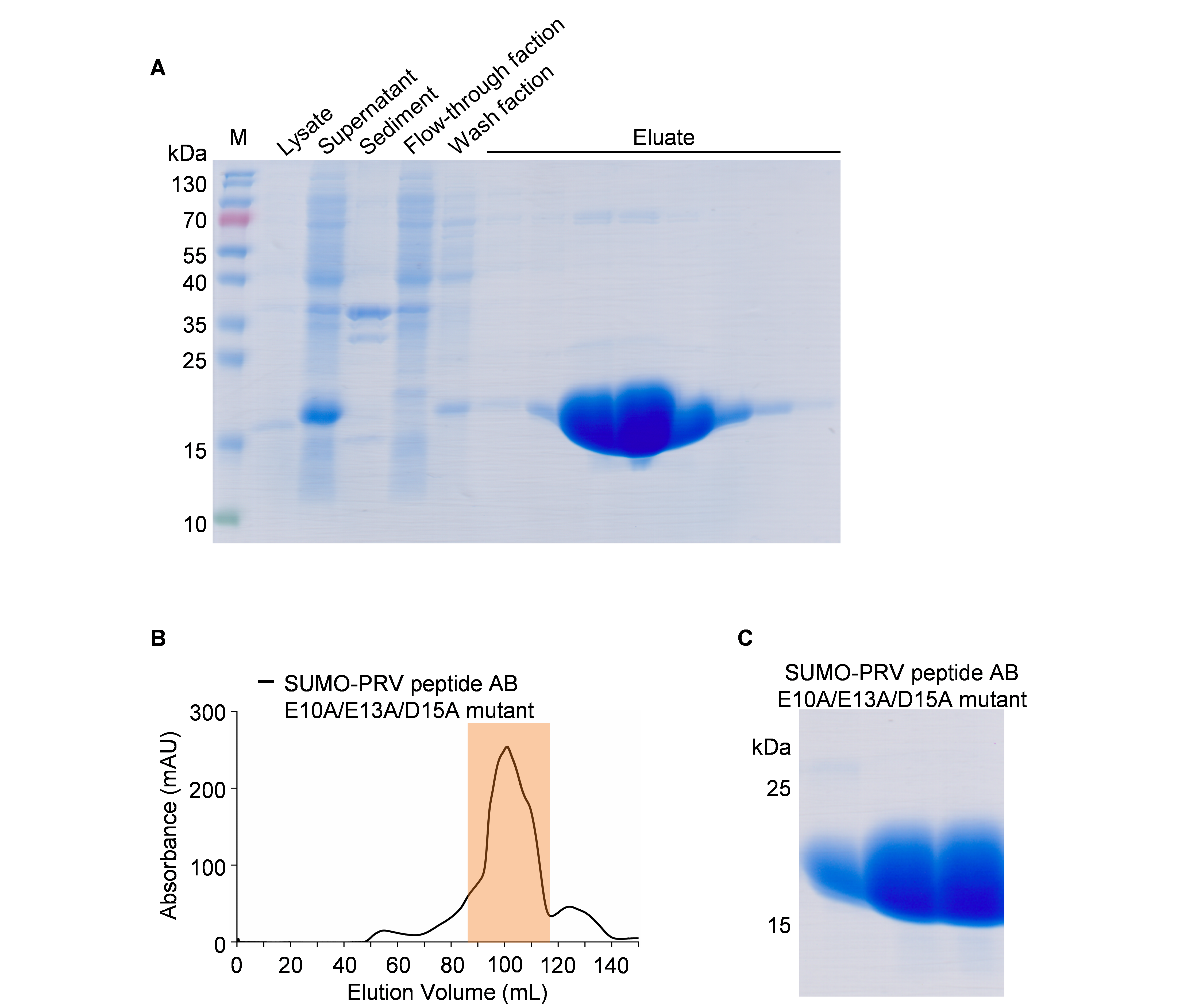

Supplement: S13 Fig — (A) Nickel column purification of SUMO-PRV peptide AB E10A/E13A/D15A mutant proteins. (B, C) The recombinant proteins SUMO-PRV US1 peptide AB E10A/E13A/D15A mutant were purified using gel filtration (B), followed by SDS-PAGE analysis (C). (TIF) [file ppat.1013669.s013.tif]

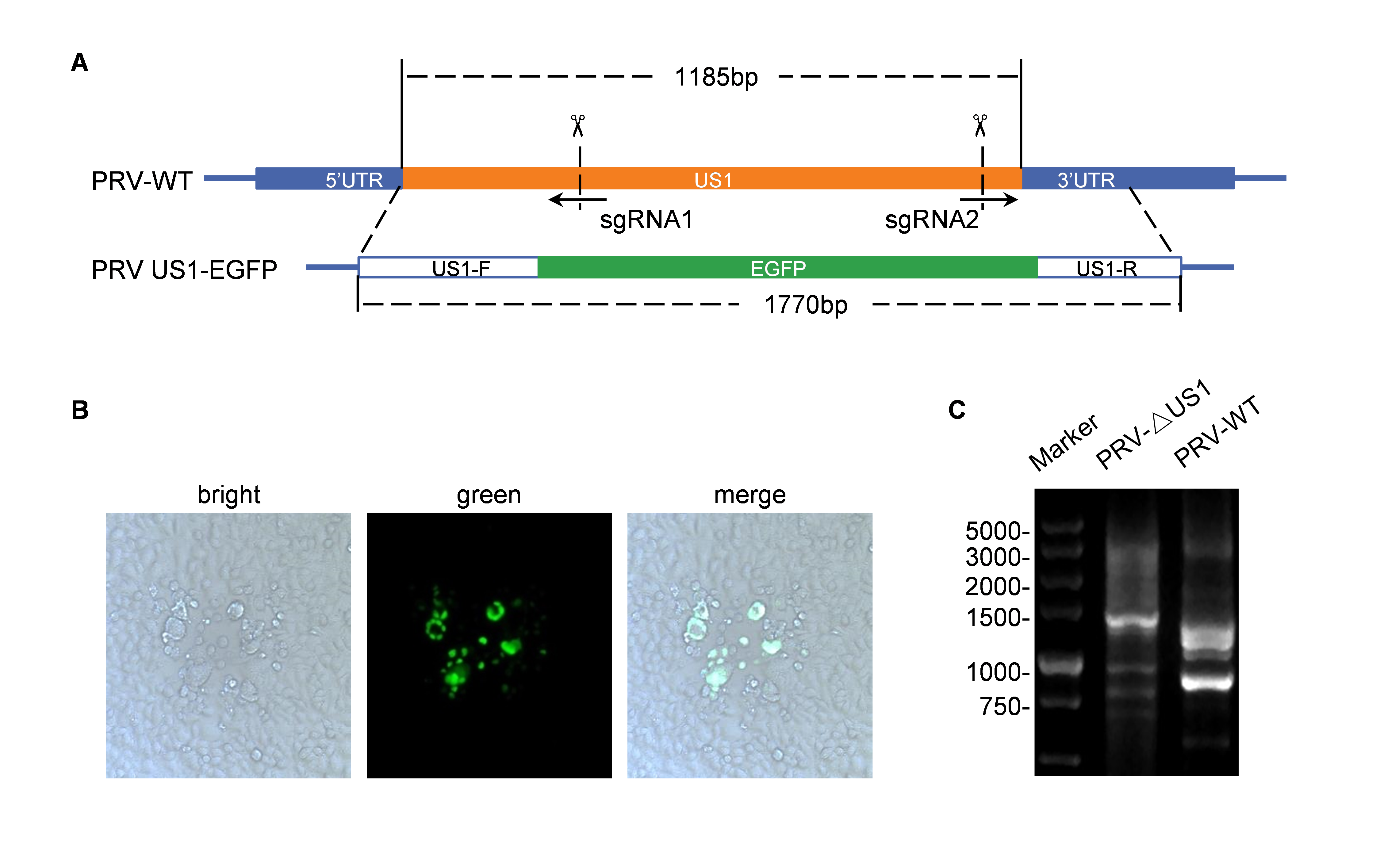

Supplement: S14 Fig — (A) Schematic illustration of strategy used to construct the PRV-ΔUS1 deletion mutant virus. (B) Monoclonal viruses expressing green fluorescence were isolated using fluorescence microscopy. (C) Verification of the US1 deletion in the mutant PRV genome was performed by PCR assay with primers designed based on the full-length US1 sequence, followed by DNA sequencing. (TIF) [file ppat.1013669.s014.tif]

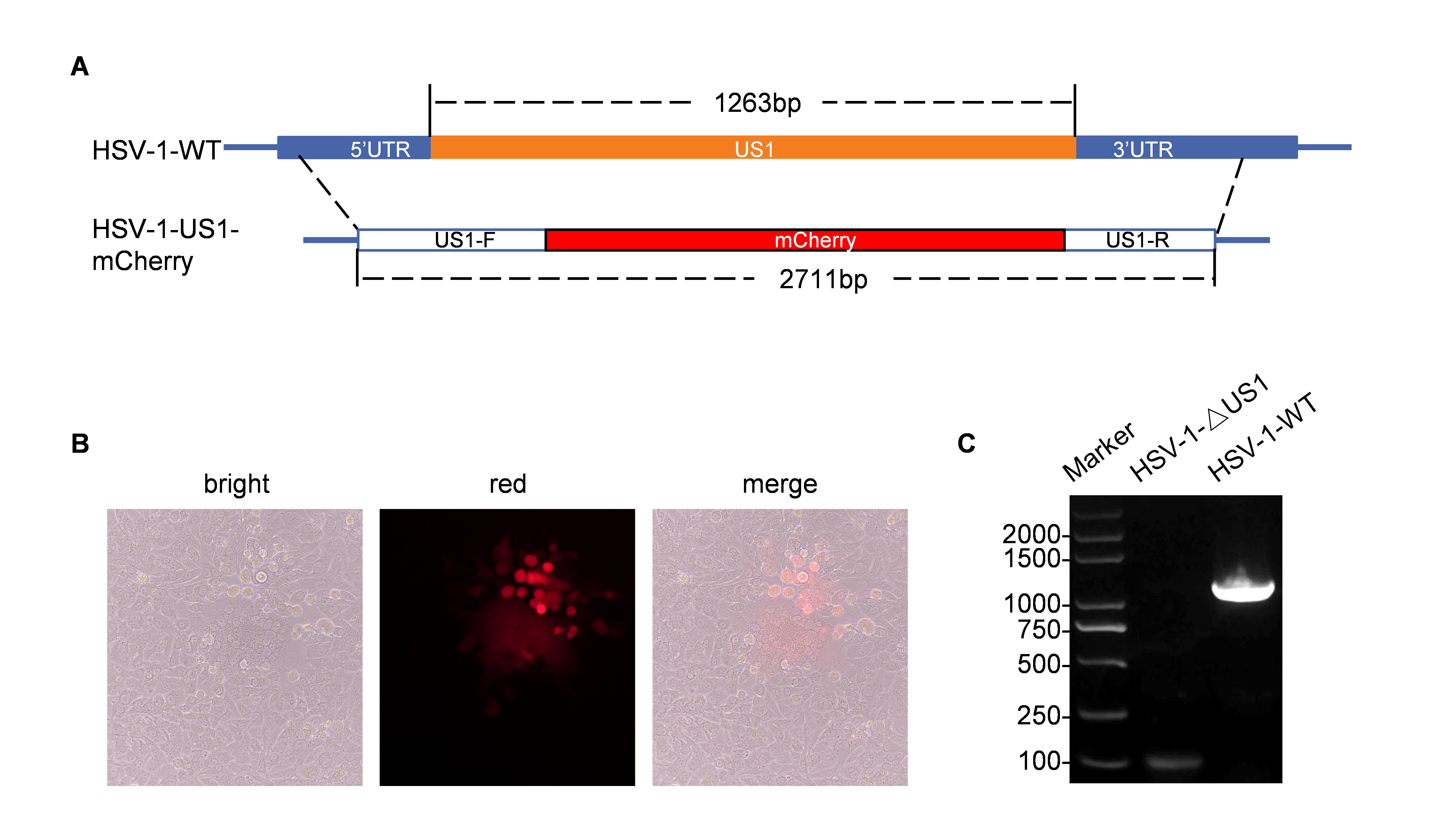

Supplement: S15 Fig — (A) Schematic illustration of the strategy used to construct HSV-1-ΔUS1 deletion mutant virus. (B) Monoclonal viruses expressing red fluorescence were identified. (C) Verification of the US1 deletion in the mutant HSV-1 genome was performed by PCR assay using primers designed based on the full-length US1 sequence. (TIF) [file ppat.1013669.s015.tif]

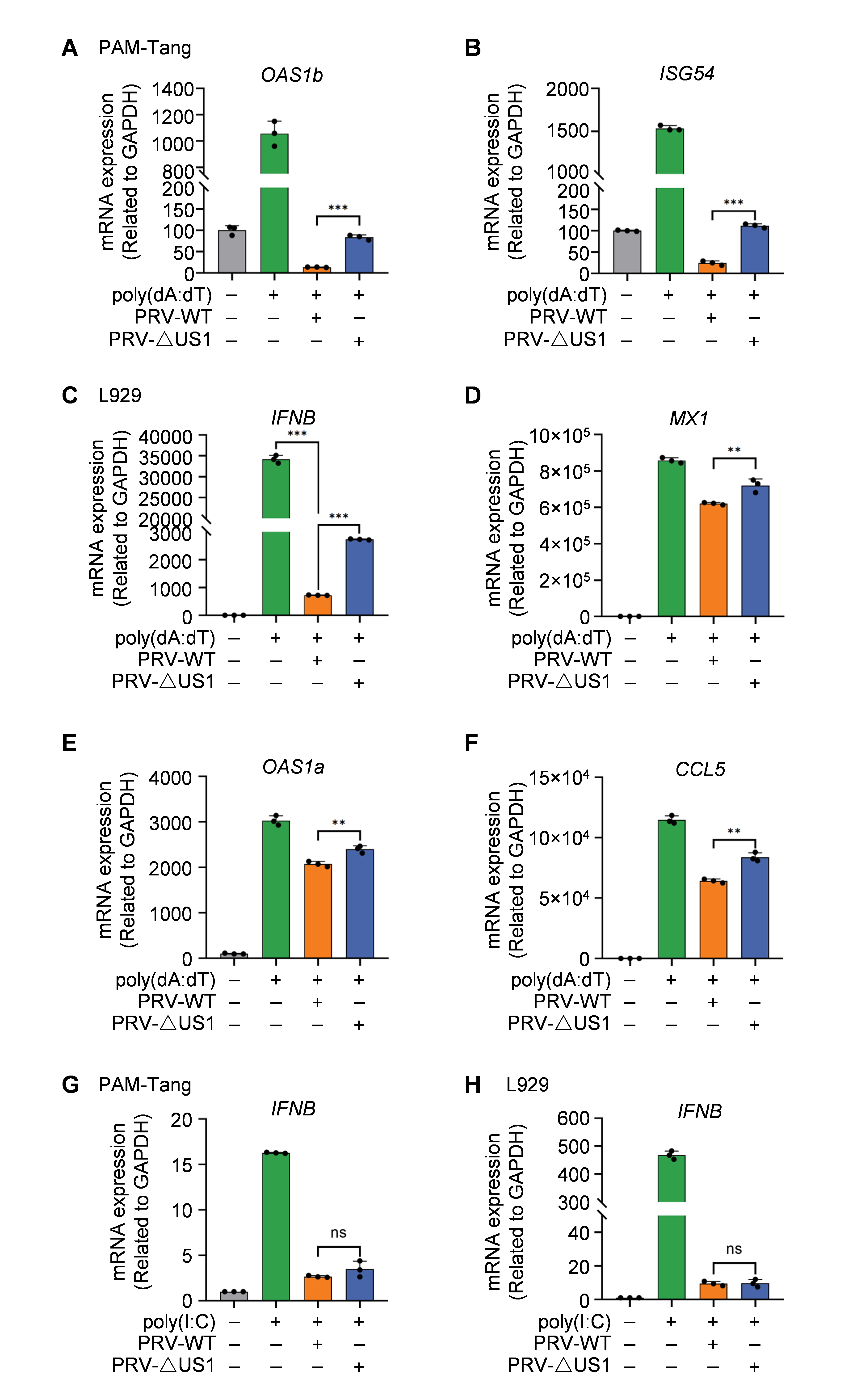

Supplement: S16 Fig — (A, B) The RT-qPCR analysis of OAS1b (A) and ISG54 (B) mRNA expression (related to GAPDH) in PAM-Tang cells infected with PRV-WT or PRV-ΔUS1 (MOI = 0.01) for 6h, followed by transfection with 1 µg poly(dA:dT) for an additional 12 hours. (C-F) The RT-qPCR analysis of IFNB (C), MX1 (D), OAS1a (E) and CCL5 (F) mRNA expression (related to GAPDH) in L929 cells infected with PRV-WT or PRV-ΔUS1 (MOI = 0.01) for 6h, followed by transfection with 1 µg poly(dA:dT) for 12 hours. (G, H) PAM-Tang or L929 cells were infected with either PRV-WT or PRV-ΔUS1 (MOI = 0.01) for 6 hours, followed by transfection with 1 µg poly(I:C) for 12 hours, the mRNA expression level of IFNB was quantified by RT-qPCR and normalized to GAPDH. (A-H) Representative results from three biological replicates are shown. Data represent mean values ± SD of three technical replicates. Statistical significance was determined by two-tailed unpaired Student’s t-tests. ***P < 0.0001, **P = 0.0089 (D), **P = 0.0041 (E), **P = 0.0010 (F). ns, not significant. (TIF) [file ppat.1013669.s016.tif]

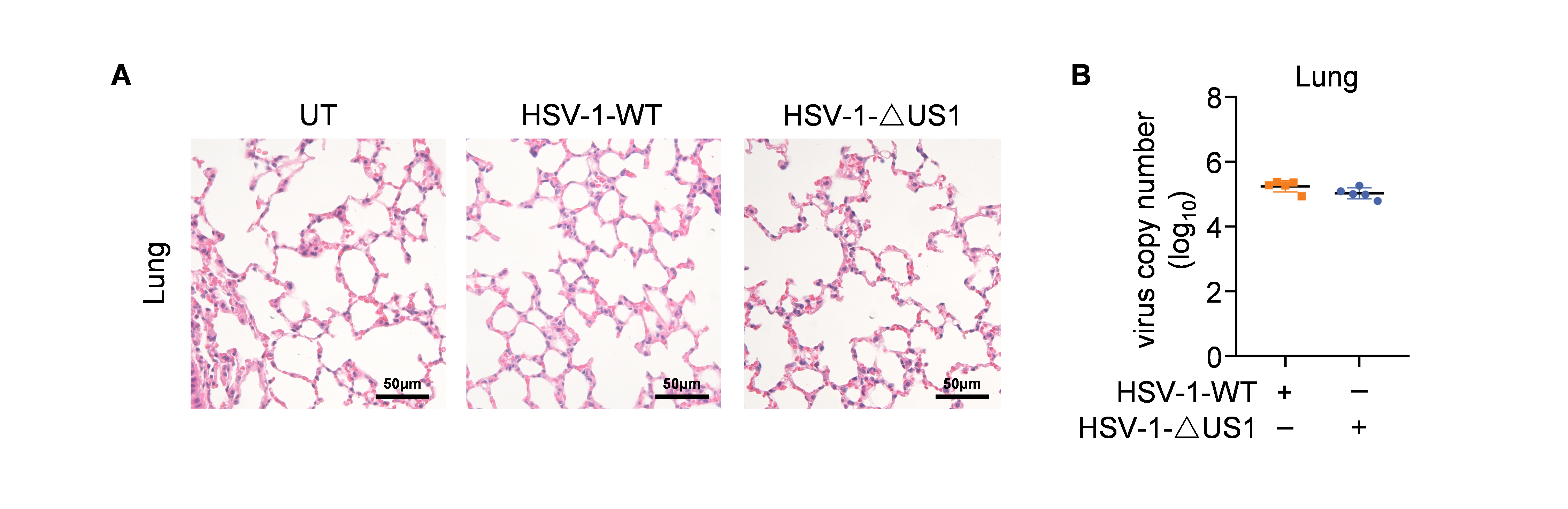

Supplement: S17 Fig — (A) H&E staining of lung tissues collected 3 days post-infection with either HSV-1-WT or HSV-1-ΔUS1 (1 × 10^7^ PFU/mouse). Representative images from five biological replicates. Scale bars, 50 μm. (B) qPCR analysis of viral replication in lung tissues from the identical infection groups shown in (A). Data represent mean values ± SD of five biological replicates. (TIF) [file ppat.1013669.s017.tif]
