# Supplementary material for: Alpha-herpesvirus US1 interacts with cGAS to suppress type I IFN responses and antiviral defense
Source: PLoS Pathog. 2025 Nov 6;21(11):e1013669. doi: 10.1371/journal.ppat.1013669 (PMC12604781; doi:10.1371/journal.ppat.1013669)
Supplement: Attachment — Submitted filename: Point-by-point reply.docx [file ppat.1013669.s019.docx]

**2025-9-11 Point-by-point reply**

 We are pleased to submit the revised manuscript entitled “**Alpha-herpesvirus US1 interacts with cGAS to suppress IFN response and antiviral defense**” for consideration in *PLoS Pathogens*. We appreciate the very positive and constructive feedback from the editorial board and reviewers. **Reviewer 1** stated: “In this manuscript, the authors reported that US1 protein from HSV-1 and PRV antagonizes the type I interferon response. US1 interacted with cGAS and inhibited its dsDNA binding and enzymatic activity. The authors further identified a conserved overlapping region in US1 from both PRV and HSV-1 that is necessary and sufficient to inhibit cGAS. For in vivo studies, US1-deficent PRV and HSV-1 induced higher innate immune response in infected mice. While the study offers new insights into innate immune evasion by alpha-herpesviruses, the conclusions would benefit from additional validation”. **Reviewer 2** noted: “In this manuscript, Qu et al. reported alpha-herpesviruses US1s have shared mechanisms to interact with and block cGAS-mediated antiviral signaling. By alpha-fold prediction, the authors also defined a structural homologous region of US1 and developed corresponding peptides which inhibited cGAS activity across species. Lastly, the authors also produced US1 deficient viruses and showed the necessary roles of US1 on viral replication and immune escape in cell culture and in mice. Overall, the study is very comprehensive with mostly convincing data. On the other hand, there are a few incoherent results or conclusions requiring attention”. **Reviewer 3** commented: “In this manuscript, the authors identified the US1 proteins of PRV and HSV-1 as inhibitors of cGAS. By AlphaFold prediction, they identified structurally conserved regions necessary for cGAS inhibition. ……Strengths: The biochemical assays demonstrating an interaction of the US1 protein with cGAS and inhibition of the cGAS-STING pathway in transfected cells are generally strong and convincing”. We have carefully addressed the reviewers’ concerns by performing new experiments to fully address the points raised. The reviewers’ questions have been highlighted in red as noted in the point-by-point reply below. We believe that the revised manuscript has addressed all concerns and that these revisions have significantly enhanced the overall quality of the manuscript.

**Reviewer 1**

Major issues：

1.The inhibition of IFN-β promoter activity by US1 shown in Figure 1, although statistically significant, lacks validation in different cell lines or experimental conditions. It is recommended to verify the inhibitory effect of US1 on the cGAS-STING signaling pathway in other relevant cell lines.

Reply: We thank the reviewer for the suggestion. In addition to experiments in HEK-293T cells, we also examined the inhibitory effect of US1 on IFN-β promoter activity in PAM-Tang cells (iPAMs, immortalized porcine alveolar macrophages). As shown in **Figure 1E**, US1 consistently suppresses IFN-β promoter activity in PAM-Tang cells, further supporting the inhibitory effect of US1 on the cGAS-STING pathway.

1500

1000

500

0

PRV US1

poly(dA:dT)

+

+

+

+

_

_

_

mRNA expression

(Related to GAPDH)

*IFNB*

PAM-Tang cells (iPAMs)

2.While the authors demonstrate that US1 interacts with cGAS, and have identified a potential region of US1 interacting with cGAS. However, further truncation of cGAS followed by interaction assays would provide more detailed molecular interaction insights.

Reply: We thank the reviewer for the suggestion. As shown in Supplementary Figure S7E, we expressed and purified recombinant PRV US1, SUMO-FLAG-pcGAS^FL^ (1-495aa), SUMO-FLAG-pcGAS^NT^ (1-134aa), and SUMO-FLAG-pcGAS^CT^ (145-495aa) proteins. We then performed pull-down assays between US1 and pcGAS^FL^, pcGAS^NT^, and pcGAS^CT^. The results show that US1 interacts with both the N-terminal and C-terminal regions of pcGAS, although these interactions are weaker than with the full-length protein, indicating that US1 engages with both ends of cGAS.

3. In FIG 4, additional cellular experiments are needed to substantiate the conclusions. For example, innate immune activation with DNA viruses or DNA mimics such as HT-DNA, followed by treatment with US1-derived peptides, would provide more compelling functional evidence.

Reply: We sincerely thank the reviewer for this insightful suggestion. To directly address this point, we have performed additional cellular experiments as recommended. Specifically, PAMs were pre-treated with the PRV US1-derived AB peptide for 6 h, followed by stimulation with poly(dA:dT) for 12 h. The mRNA levels of *Ifnb*, *Isg15* and *Isg54* were significantly reduced compared to the control group, confirming the inhibition of IFN-I by PRV AB peptide (Fig. **4M**). This inhibitory effect was further verified by the HSV-1 US1 AB peptide (Fig. **4N**). Together, these results provide functional evidence that the US1-derived peptide effectively inhibits cGAS-mediated innate immune activation both in the recombinant protein and cellular levels, ultimately leading to the suppression of IFN-I production.

**Fig 4** (M, N) PAMs (M) or HeLa cells (N) were treated with AB peptides derived from PRV US1 or HSV-1 US1 for 6 hours, followed by stimulation with poly(dA:dT) for 12 hours. Cells were then collected, RNA was extracted, and qPCR was performed to assess gene expression.

4.In figure 5, Although the authors have observed a significant increase in IFN-β production upon infection with US1-deficient viruses (PRV-ΔUS1 and HSV-1-ΔUS1), they have not assessed how US1 deficiency affects viral replication. Does US1 deficiency lead to significant attenuation of viral replication? Furthermore, whether the deletion of key innate immune components (e.g., TBK1 or IRF3) at least partially rescue the replication of US1-deficient viruses? Addressing these questions would clarify whether the enhanced immune response is causally linked to US1's immune antagonism.

Reply: Thank you for your valuable suggestion. To further address this issue, we compared the replication of PRV WT and PRV ΔUS1 in primary porcine alveolar macrophages (PAMs) and examined the effect of cGAS knockdown on PRV ΔUS1 replication. Specifically, PAMs (WT or cGAS knockdown) were infected at an MOI of 0.01, and cell supernatants were collected at 12, 24, 36, and 48 hours post infection. Viral DNA was extracted and quantified by absolute qPCR. As shown in the results, in WT PAMs, the viral DNA copy number of PRV WT was significantly higher than that of PRV ΔUS1. However, in cGAS-deficient PAMs, the viral DNA copy number of PRV ΔUS1 was almost restored to the level observed for PRV WT in PAMs. Together with our previous finding that US1-deficient viruses induce markedly elevated IFN-β production, these data indicated that the reduced replication capacity of PRV ΔUS1 is largely due to enhanced interferon-mediated antiviral restriction. Importantly, when cGAS was silenced, IFN-I induction was impaired, and the replication of PRV ΔUS1 was rescued to WT levels, further demonstrating that US1 plays a critical role in antagonizing cGAS-mediated IFN-β production.

WT PAMs were infected with PRV WT or PRV ΔUS1 at an MOI of 0.01, while cGAS-knockdown PAMs were infected with PRV ΔUS1 at the same MOI. Cell supernatants were collected at 12, 24, 36, and 48 h post infection, and viral DNA was extracted for absolute quantitative PCR analysis.

5. The in vivo experiments in FIG 6 primarily measured IFN-β levels and viral load. However, the results may not specifically reflect the antagonization of cGAS by US1, considering the multifaceted functions of US1. Since the authors have defined the cGAS interacting region of US1 in Figure 3. The authors may consider generating a mutant HSV-1 with selective disruption of this interaction (e.g., point mutations in the binding domain) rather than a complete US1 knockout. Although not perfect, such a mutant virus would provide a more precise tool to evaluate the immunomodulatory role of US1 both in vitro and in vivo.

Reply: We thank the reviewer for the suggestion. Previous studies have reported that HSV-1 UL21 degrades cGAS through the autophagy–lysosome pathway, and in those studies, only deletion viruses were generated to demonstrate cGAS antagonism (Ma et al., Autophagy, 2023, PMID: 36343628). Similarly, KSHV ORF52 was shown to antagonize cGAS by blocking its binding to DNA, and only a VP22 deletion virus was constructed in that work (Wu et al., Cell Host & Microbe, 2015, PMID: 26320998). In other words, these herpesviruses relied solely on deletion viruses to validate the mechanism of cGAS antagonism. In our own attempts, we generated multi-site alanine mutants in the A, B, and AB regions of US1 to substitute for US1 ΔA, ΔB, and ΔAB; however, these multi-point mutants failed to express properly. Therefore, we believe that the combined evidence from deletion virus-based cellular and animal experiments, together with biochemical assays, sufficiently supports the conclusion that US1 antagonizes cGAS.

Minor issues:

1.The phrase “overlapping peptides within PRV and HSV-1 US”, in the abstract and elsewhere, is misleading. Since the identified region is part of the US1 protein and not a naturally processed peptide, it would be more accurate to refer to it as a “conserved overlapping region”.

Reply: Thank you for your valuable suggestion. “Structural analysis using AlphaFold reveals overlapping peptides within PRV and HSV-1 US1 proteins.” was replaced by “Structural analysis using AlphaFold reveals a conserved overlapping region within PRV and HSV-1 US1 proteins.”

2.Similarly, line 102-103, the phrase “utilizing viral peptides to antagonize cGAS detection” is also inappropriate. This region is not a peptide generated during viral infection, while the use of synthetic peptides in experimental assays is acceptable.

Reply: Thank you for your valuable suggestion. “These findings uncover a novel strategy by which alpha-herpesviruses counteract host innate immunity, utilizing viral peptides to antagonize cGAS detection, thereby illustrating a sophisticated mechanism of immune evasion.” was replaced by “These findings uncover a novel strategy by which alpha-herpesviruses counteract host innate immunity, utilizing viral US1 protein antagonizes cGAS recognition, with its structurally conserved region playing a critical role, thereby illustrating a sophisticated mechanism of immune evasion.”

3. The Materials and Methods section should include information on the primers used in the study.

Reply: Thank you for your kind reminder. We have included the primer information in Table 1.

**Reviewer #2:**

Major issues：

(1) HSV-2 US1 has been reported to block IRF3 for IFN-I inhibition. Another study also shows that HSV-1 US1 blocks STING-induced interferon responses (PMCID: PMC6854482). The authors should discuss these discrepancies. Moreover, for Figure 1 most of the reporter assays were done in both cGAS and STING overexpression manner. The authors should try STING overexpression along with US1 to probe whether US1 also inhibits STING-mediated responses.

Reply: Thank you for your valuable suggestion. “The US1 protein of herpesviruses has also been reported to antagonize the interferon pathway, among which HSV-2 immediate-early protein US1 downregulates IFN-β production by inhibiting the interaction of IRF-3 with the IFN-β promoter, revealing an unconventional mechanism by which HSV-2 evades host innate immunity[38]. HSV-1 US1 can inhibit STING-induced IFN-β activation[39]; however, our study primarily focuses on US1 antagonism of cGAS, which was added in discussion. Since the focus of our study is on US1 antagonism of cGAS, we did not assess whether US1 affects STING or IRF3.

(2) (Line 153) It is confusing to me as the author could readily express Flag-tagged HSV-1 US1 but fail to ‘express’ and ‘purify’ the protein. As VZV US1 did not demonstrate optimal anti-cGAS activities compared to PRV US1 (Figures 2H and 2J), purified HSV-1 US1, if possible, could provide a much stronger inhibitory effect.

Reply: Thank you for your comment. We were able to express HSV US1-Flag in HEK-293T cells; however, it is unsuccessful to express HSV US1 in *E*.coli. We also tried alternative vectors and expression strategies, but HSV US1 could not be expressed in these systems.

Alpha-herpesvirus, VZV US1 also significantly suppressed cGAS-STING induced interferon reporter activity (Figure 1K). Alternatively, we expressed and purified the VZV US1 in *E*.coli (Figure S3C-S3E). Purified VZV US1 still impaired the 2’,3’-cGAMP production, although not stronger than PRV US1, indicating the broad inhibitory impact of cGAS among different types of alpha-herpesvirus.

(3) Figure 2E, it seemed that the overexpressed cGAS and US1 were mutually exclusive in the nucleus, which did not substantially support the authors’ conclusion on colocalization; moreover, Figure 2F was also weak provided the fact that both proteins demonstrated whole cytoplasmic distribution. It is better to overexpress US1 in a cell line with good signals for endogenous cGAS and also to perform quantification of the signal overlapping percentage.

Reply: We thank the reviewer for this helpful suggestion. To address this concern, we quantified fluorescence intensity using ImageJ. The results showed that PRV US1 and cGAS displayed nearly identical fluorescence intensity profiles. For VZV US1 and cGAS overexpression, we collected three independent datasets, all of which consistently demonstrated clear colocalization between US1 and cGAS.

(4) Figure S7E, if PRV US1 had significantly reduced binding to both pcGAS NT and CT, how did it block NT and CT from binding to dsDNA (Figures S7B and S7C)?

Reply: We thank the reviewer for this helpful suggestion. Although the interaction of US1 with either the N- or C-terminal region of cGAS is weaker than with the full-length protein, detectable binding is still observed, which suggests that both the N- and C-terminal regions of cGAS provide interacting sites, but each region alone offers fewer sites, resulting in weaker interactions compared to the full-length protein. US1 interacts with cGAS and inhibits its binding to dsDNA. Consistently, the results shown in **Figures S7B-D** demonstrated that US1 suppressed the binding of both the N- and C-terminal regions of cGAS to dsDNA.

(5) Figure 2J is hard to interpret as VZV US1 inhibited the formation of cGAS-dsDNA complex but did not result in more naked dsDNA in the gel.

Reply: Thank you for your comment. We performed densitometric analysis (ImageJ) of the free DNA and found that VZV US1 still increases the amount of free DNA.

(6) Figure 3C and 3D need protein expression immunoblots as expression levels of the mutants determine their potency in antagonizing cGAS.

Reply: We thank the reviewer for this helpful suggestion. We have added Western blot analysis of protein expression in Fig. 3C and 3D.

(7) Figure 3G showed a much broader peak for cGAMP which was not observed in any other cGAS activity assay results.

Reply: We thank the reviewer for this helpful suggestion. In the previous cGAS activity assay results, the peak of PRV US1 was relatively broad, which might have been due to decreased column efficiency of MonoQ after repeated usage. After cleaning the MonoQ column with 0.5 M NaOH, we repeated the cGAS activity assay. PRV US1 markedly suppressed cGAS activity (red line).

Fig3.G

(8) The negatively charged residues ‘EED’ are not present in the proposed overlapping region of HSV-1 US1. Thus, the models predicted by Figures 4M and 4N may not apply to HSV-1 US, which weakens the hypothesis that this aligned region is the conserved structural component inhibiting cGAS.

Reply: We appreciate the reviewer’s insightful comment. Indeed, the negatively charged residues E10, E13, and D15 (“EED”), which mediate the interaction of PRV US1 peptide AB with cGAS, are not present in the corresponding aligned region of HSV-1 US1. In our study, although we were unable to purify HSV-1 US1 protein for direct biochemical validation, our cellular assays demonstrated that HSV-1 US1 and its derived peptide AB are also capable of suppressing cGAS-mediated IFN-I induction. Therefore, while the structural model proposed in Figures 4M and 4N may not fully apply to HSV-1 US1, we speculate that the conservation lies in the functional outcome (cGAS inhibition) rather than in identical structural determinants. We have clarified this limitation in the revised manuscript and highlighted the possibility that HSV-1 US1 employs an alternative structural interface to inhibit cGAS.

Minor issues:

(1) Line 144 (Figure 1L), as the basal level of IFN-beta was low in HSV-1-infected cells, it is not strong evidence to claim that US1 fails to suppress IFN-beta in the absence of cGAS/STING overexpression.

We thank the reviewer for this helpful suggestion. “The results showed that, in contrast to the inhibitory effect observed with the addition of cGAS and STING, neither PRV nor HSV-1 US1 alone was capable of suppressing IFN-β promoter activity (Fig 1L).” was replaced by “The results showed that, in contrast to the inhibitory effect observed with the addition of cGAS and STING, neither PRV nor HSV-1 US1 alone was capable of activating IFN-β promoter activity (Fig 1L).”

(2) Line 244, as all three PRV US1 mutants demonstrated partial inhibition on cGAS to bind dsDNA (Figure 3H), the conclusion that the mutants did not exhibit reduction is inaccurate.

Reply: Thank you for your suggestion. We have revised the inaccurate statement. “Consistent with these findings, PRV US1 protein significantly reduced pcGAS binding to ISD45 DNA, whereas the three deletion mutants did not exhibit this reduction (Fig 3H).” was replaced by “Consistent with these findings, PRV US1 protein significantly reduced pcGAS binding to ISD45 DNA, whereas the inhibitory effect was partially diminished in the three deletion mutants (Fig 3H).”

(3) Without a linker sequence between peptide A and B, it would be challenging to adapt to a kinked helix structure as presented in Figure 3A. As such, the contribution of A in the peptide AB may not reflect or mimic what truly happens in the context of the whole protein. The authors should consider this possibility when describing the results.

Reply: Thank you for your careful suggestion. Although the actual structures of peptide AB and of A and B individually remain undetermined, this modeled schematic helps readers better visualize and understand our study.

(4) It seems that peptide AB blocked cGAS binding to dsDNA at a much lower efficiency compared to the full protein (Figure 4D). Nevertheless, it potently inhibited cGAMP production. According to Figure M and N, the peptides could interact with cGAS at two different sites, which may explain their potency in antagonizing cGAS activity. However, in the context of a full protein, it is unlikely that both mechanisms exist considering the steric hindrance of protein-protein interactions. The authors should discuss this point when describing the result.

Reply: We thank the reviewer for this helpful suggestion. “The actual conformations of peptides AB, A, and B remain unknown and may differ from those in the full-length protein. Nevertheless, we have demonstrated the antagonistic effect of these peptides on cGAS through in vitro biochemical assays and cellular experiments.” was added in discussion.

**Reviewer #3:**

Major issues：

1. The introduction mentions alphaherpesvirus genes and proteins inhibiting the cGAS-STING signaling pathway without describing their mechanism of action. This information is important for the understanding of the present study. It is important to understand whether the different inhibitors function in a complementary or redundant fashion. It is also necessary to describe other known functions of US1 in the introduction (not only in the discussion).

Reply: Thank you for your valuable suggestion. “UL21[11] antagonizes IFN-β production by promoting cGAS degradation through the autophagolysosomal pathway, while VP22[24] inhibits cGAS by disrupting cGAS-DNA phase separation.” was added in introduction.

2. Figure 5. Several PRV and HSV-1 proteins target the cGAS-STING pathway, suggesting that they have redundant functions. Why is the deletion of US1 sufficient to activate the pathway in infected cells? Do the ΔUS1 viruses replicate to lower titers in THP-1 (or other) cells, and can the replication defect be rescued by knocking out of cGAS or STING?

Reply: Thank you for your valuable suggestion. To further address this issue, we compared the replication of PRV WT and PRV ΔUS1 in primary porcine alveolar macrophages (PAMs) and examined the effect of cGAS knockdown on PRV ΔUS1 replication. Specifically, PAMs (WT or cGAS knockdown) were infected at an MOI of 0.01, and cell supernatants were collected at 12, 24, 36, and 48 hours post infection. Viral DNA was extracted and quantified by absolute qPCR. As shown in the results, in WT PAMs, the viral DNA copy number of PRV WT was significantly higher than that of PRV ΔUS1. However, in cGAS-deficient PAMs, the viral DNA copy number of PRV ΔUS1 was almost restored to the level observed for PRV WT in WT PAMs. Together with our previous finding that US1-deficient viruses induce markedly elevated IFN-β production, these data indicate that the reduced replication capacity of PRV ΔUS1 is largely due to enhanced interferon-mediated antiviral restriction. Importantly, when cGAS was silenced, IFN-I induction was impaired, and the replication of PRV ΔUS1 was rescued to WT levels, further demonstrating that US1 plays a critical role in antagonizing cGAS-mediated IFN-β production.

WT PAMs were infected with PRV WT or PRV ΔUS1 at an MOI of 0.01, while cGAS-knockdown PAMs were infected with PRV ΔUS1 at the same MOI. Cell supernatants were collected at 12, 24, 36, and 48 h post infection, and viral DNA was extracted for absolute quantitative PCR analysis.

3. Figure 6. The ΔUS1 viruses are attenuated in vivo. However, considering that US1 has additional functions, it is not clear whether the attenuation is caused by a lack of cGAS inhibition. This could be addressed by using a virus expressing a mutant US1 protein that is unable to inhibit cGAS but retains other US1 functions. Alternatively, the authors could demonstrate a (partial) rescue of ΔUS1 viruses in cGAS or STING KO mice.

Reply: We thank the reviewer for the suggestion. Previous studies have reported that HSV-1 UL21 degrades cGAS through the autophagy–lysosome pathway, and in those studies, only deletion viruses were generated to demonstrate cGAS antagonism (Ma et al., Autophagy, 2023, PMID: 36343628). Similarly, KSHV ORF52 was shown to antagonize cGAS by blocking its binding to DNA, and only a VP22 deletion virus was constructed in that work (Wu et al., Cell Host & Microbe, 2015, PMID: 26320998). In other words, these herpesviruses relied solely on deletion viruses to validate the mechanism of cGAS antagonism. In our own attempts, we generated multi-site alanine mutants in the A, B, and AB regions of US1 to substitute for US1 ΔA, ΔB, and ΔAB; however, these multi-point mutants failed to express properly. Therefore, we believe that the combined evidence from deletion virus-based cellular and animal experiments, together with biochemical assays, sufficiently supports the conclusion that US1 antagonizes cGAS.

4. Lines 246-252. It is very confusing that alanine substitution mutants behaved essentially like WT US1. Doesn’t this challenge the importance of the peptides?

Reply: We thank the reviewer for the comment. As shown in Figures 3I–J, we generated PRV US1 A, B, and AB alanine substitution mutants and found that these mutants lost the ability to antagonize cGAS. This indicates that the A, B, and AB regions are critical for US1-mediated inhibition of cGAS.

Minor issues:

 1. Line 63. “genital infections” rather than “reproductive system infections”.

Reply: We thank the reviewer for the suggestion. In line 63, “reproductive system infections” was replaced by “genital infections”

2. Line 108. Inhibition of the cGAS-STING pathway by alphaherpesvirus proteins has been shown before. Hence, the authors did not uncover a novel strategy to counteract innate immunity.

Reply: We thank the reviewer for the suggestion. “a novel strategy” was replaced by “a conserved strategy”.

3. Fig. 1A and B. There are no positive controls.

Reply: We thank the reviewer for the comment. In Fig. 5F-J, poly(dA:dT) stimulation group was used as a positive control to confirm the responsiveness of the assay system. In Fig. 1A and 1B, however, our aim was not to present positive controls but rather to demonstrate that, despite being DNA viruses, PRV and HSV-1 failed to induce IFN-β expression in PK-15 and HeLa cells. This unexpected lack of induction suggests that these viruses may harbor specific mechanisms to suppress IFN-I signaling.

4. Line 169. Interacted endogenously with pcGAS -> interacted with endogenous pcGAS

Reply: We thank the reviewer for the suggestion. “Interacted endogenously with pcGAS” was replaced by “interacted with endogenous pcGAS”

5. Line 172-173 and Figs. 2E and F. Colocalization is not convincing. Both proteins are cytoplasmic.
Reply: We thank the reviewer for this helpful suggestion. To address this concern, we quantified fluorescence intensity using ImageJ. The results showed that PRV US1 and cGAS displayed nearly identical fluorescence intensity profiles. For VZV US1 and cGAS overexpression, we collected three independent datasets, all of which consistently demonstrated clear colocalization between US1 and cGAS.

6. Line 347. we challenged L929 cells with HSV-1-ΔUS1 or HSV-1-WT -> we infected

Reply: We thank the reviewer for the suggestion. “we challenged L929 cells with HSV-1-ΔUS1 or HSV-1-WT” was replaced by “we infected L929 cells with HSV-1-ΔUS1 or HSV-1-WT”.

7. Discussion, line 418-425. Delete this part as it is not relevant for the present study.

Reply: We thank the reviewer for the suggestion. This part was delated.
